# Supplementary material for: New Insights into Boron Essentiality in Humans and Animals
Source: Int J Mol Sci. 2022 Aug 15;23(16):9147. doi: 10.3390/ijms23169147 (PMC9409115; doi:10.3390/ijms23169147)
Supplement: Supplementary file 1 [file ijms-23-09147-s001.zip › ijms-1823150-supplementary.pdf]

SUPPLEMENTARY MATERIAL

PART S1

In vivo calcium fructoborate (CaFB) feeding

In vivo CaFB feeding was carried out on two groups of six months old male Wistar rats, each of two animals, with an average weight of 290±10 g:

• Group 1 (B-supplemented) → two rats fed with CaFB as 150 ppm B;

• Group 2 (Reference) → two rats fed the normal diet.

During the first day of the experiment, the rats were chosen and assigned a specific group, while the second day each group started to receive the high boron diet (Group 1) and the normal diet (Group 2), respectively.

Throughout the experiment, animals were kept under observation in individual cages, in the Animal Facility of the University of Medicine and Pharmacy of Craiova, Romania, under standard conditions of temperature, humidity, lighting (12-hour light/dark cycle – 7/7), food and water (*ad libitum*).

The next seven days, fecal matter was collected from each rat and food was resupplied at four days.

At the end of the seven days, the rats were euthanized, under general anesthesia, and the following parts were stored for later analysis: (*i*) cecum mucus, (*ii*) colon mucus, (*iii*) rectum with mucus.

The general anesthesia was performed by intramuscular injection of an anesthetic cocktail consisting of Ketamine hydrochloride 90 mg/kg body weight (b.w.) (Ketamidor® 100 mg/mL, Richter Pharma AG, Austria) and Xylazine hydrochloride 10 mg/kg b.w. (Xylazin Bio® 2%, Bioveta, Czech Republic).

The experimental protocol was applied according with the European Council Directive No. 86/609 (November 24, 1986), the European Convention for the Protection of Vertebrate Animals used for Experimental and Other Scientific Purposes (December 2, 2005), and the Romanian Parliament Law No. 43 (April 11, 2014) on the protection of animals used for scientific purposes. The study was approved by the Ethics Committee of the University of Medicine and Pharmacy of Craiova (Approval No. 51/20.04.2018).

PART S2

In vitro digestion simulation

In vitro digestion simulation was performed using a gastric simulator adapted after Ferrua & Singh (2015) [142], Wang et al. (2021) [143] and Kong & Singh (2010) [144].

Simulated gastric fluid (SGF) was prepared by dissolving 1.38 g NaCl, 0.0612 g KH_2PO_4, 0.257 g KCl, 0.0122g $MgCl_2$, 1.05 g $NaHCO_3$ and 0.024 g $(NH_4)_2CO_3$ per 0.5 L of SGF, adjusting the pH with 0.6 N HCl. $CaCl_2$·• H_2O (0.0193 g/0.5 L of SGF) was not added to the SGF solution initially, as it may precipitate, but was added to the final mixture after adjusting the pH. At the final mixture, we added 2.5 g porcine pepsin to simulate enzymatic activity [145].

The experiment was conducted at three distinct pH values, knowing that SGF has different pH values, usually with values between 3–5, depending on the type of food consumed, or below 1.5 on an empty stomach: pH 1.3, to simulate fasted gastric pH, which is commonly below 1.5 [146]; pH 3, when the postprandial pH was simulated in the case of a typical meat and cola meal; pH 4.5, when the postprandial pH was simulated in the case of a typical meal with vegetables.

Gastric motility was stimulated using a shaker at 100 rpm (stimulating actual stomach contraction). To stimulate the preprandial environment of the stomach (before a meal), 70 mL of simulated gastric juice was first placed in the gastric simulator and brought to 37°C. 500 mg of Fructoborate (FB) was added over it, and an initial FB sample of 2 mL was

taken (7.14 mg/mL FB). The addition of FGS was started for three hours at a flow rate of 2.5 mL/min. At regular intervals (30, 60, 90, 120, 150, 180 minutes), equal amounts (2 mL) of solution were extracted and subjected to physicochemical analysis. All experiments were performed in triplicate.

Table S1. Parameters for in vitro dynamic gastric digestion

**Gastric conditions (37°C)**	
Simulated gastric fluid (SGF)	Constituent concentration in SGF [mmol/L]
NaCl	47.2
KH_2PO_4	0.9
KCl	6.9
$MgCl_2 \cdot 6H_2O$	0.12
$(NH_4)_2CO_3$	0.5
$CaCl_2 \cdot H_2O$	0.14
$NaHCO_3$	25
Enzyme	Concentration [g/L]
Porcine pepsin	2.5
pH	1.3, 3, 4.5
Gastric motility	100 rpm
Digestion time	180 minutes
Flow rate	2.5 mL/min

Table S2. Measurements of fructoborate (FruitexB) in vitro gastric digestion

**Digestion time [min]**	**% Fructoborate in gastric chyme**											
	pH 1.3			*±SD*	***pH 3***			*±SD*	***pH 4.5***			*±SD*
	Ist Dtn.	*IInd Dtn.*	*IIIrd Dtn.*		*Ist Dtn.*	*IInd Dtn.*	*IIIrd Dtn.*		*Ist Dtn.*	*IInd Dtn.*	*IIIrd Dtn.*	
30	63.34	68.23	70.15	2.87	88.32	83.12	83.02	2.48	97.05	93.45	96.95	1.67
60	69.45	63.67	68.54	2.54	89.03	89.12	84.78	2.03	97.67	93.94	94.38	1.66
90	65.78	64.08	67.11	1.24	87.28	83.49	85.11	1.55	97.07	94.04	93.19	1.67
120	68.21	67.07	65.56	1.09	85.29	84.67	86.38	0.71	94.74	97.12	94.76	1.12
180	65.45	59.83	61.73	2.33	78.59	83.30	80.09	1.96	87.11	94.23	87.37	3.30

Dtn.: Determination; SD: Standard deviation.
